# Novel frameshift variant in the *PCNT* gene associated with Microcephalic Osteodysplastic Primordial Dwarfism (MOPD) Type II and small kidneys

**DOI:** 10.1186/s12920-022-01226-8

**Published:** 2022-04-14

**Authors:** D. Hettiarachchi, S. M. V. Subasinghe, G. G. Anandagoda, Hetalkumar Panchal, P. S. Lai, V. H. W. Dissanayake

**Affiliations:** 1 Department of Anatomy, Genetics and Biomedical Informatics, Faculty of Medicine, University of Colombo, Colombo, Sri Lanka; 2 Lady Ridgeway Hospital for Children, Colombo, Sri Lanka; 3 Post Graduate Department of Bioscience, Sardar Patel University, Vallabh Vidyanagar, Gujarat India; 4 Department of Paediatrics, Yong Loo Lin School of Medicine, National University of Singapore, Singapore, Singapore

## Abstract

**Background:**

Microcephalic Osteodysplastic Primordial Dwarfism (MOPD) Type II is an autosomal recessive condition encompassing a heterogeneous group of disorders characterized by symmetrical growth retardation leading to dwarfism, microcephaly, and a range of multiple medical complications including neurovascular diseases. Biallelic pathogenic variants in the pericentrin gene (*PCNT*) have been implicated in its pathogenesis.

**Case presentation:**

We performed whole-exome sequencing to ascertain the diagnosis of a 2 year and 6 months old boy who presented with severe failure to thrive, microcephaly, and facial gestalt suggestive of MOPD Type II which included features such as retrognathia, small ears, prominent nasal root with a large nose, microdontia, sparse scalp hair, bilateral fifth finger clinodactyly. He had a small ostium secundum atrial septal defect and bilaterally small kidneys. Microcephalic Osteodysplastic Primordial Dwarfism (MOPD) Type II was confirmed based on a pathogenic compound heterozygous frameshift variant in the *PCNT* gene c.5059_5060delAA | p. Asn1687fs (novel variant) and c.9535dup (p. Val3179fs). His parents were found to be heterozygous carriers for the variants.

**Conclusion:**

We report a novel frameshift variant in the *PCNT* gene and a previously unreported phenotype for Microcephalic Osteodysplastic Primordial Dwarfism (MOPD) Type II.

## Background

Microcephalic Osteodysplastic Primordial Dwarfism (MOPD) Type II (OMIM #210720) is a clinically heterogeneous group of conditions characterized by both pre and post-natal growth retardation together with microcephaly. This condition was first described in 1982 by Majewski Ranke, and Schinzel [1]. Described under the umbrella of Primordial dwarfism (PD) which comprises of several subtypes: Seckel syndrome, Russell Silver syndrome, Meier-Gorlin syndrome, and Majewski Osteodysplastic Primordial Dwarfism (MOPD) I/III and III. Currently, MOPD Type II is known to be the most common subtype. It is inherited as an autosomal recessive disorder caused by biallelic loss of function mutations in the pericentrin (*PCNT*) gene [2, 3]. Individuals who suffer from this condition have characteristic facies which include a prominent nose and disproportionate features, skeletal dysplasia, impaired growth persisting throughout the post-natal period reaching stunted adult size (average height of 40 cm post-pubertal and adult height of under 100 cm), abnormal dentition and insulin resistance [4, 5]. The care of these patients has now advanced owing to the increased accessibility of high throughput sequencing technologies such as Next Generation Sequencing. A more proactive approach can be taken to address their orthopedic manifestations, insulin resistance, hematological abnormalities, susceptibility to neurovascular diseases including systemic hypertension and renal complications. Thus, they should be encouraged to undergo regular screening to prevent cerebrovascular disease and growth monitoring [2, 6]. Even though MOPD Type II is associated with smaller brain sizes than average, their IQ is near normal [7]. The pericentrin (*PCNT*) gene located on chromosome 21q22.3 is implicated in mitotic spindle formation and chromosomal segregation [8]. The pericentrin protein (~ 370 kD), encoded by this gene is an anchoring protein that binds to calmodulin expressed in centrosomes. Additionally, it is a cell cycle regulator. The protein consists of a series of highly conserved coiled-coil domains. Thus far 41 pathogenic variants and 3 likely pathogenic variants are reported in Clinvar [9]. In this study, we describe a novel compound heterozygous variant in *PCNT* gene c.9535dup (p. Val3179fs), c.5059_5060delAA (p. Asn1687fs) giving rise to a new phenotype in a baby of Sri Lankan origin.

## Case presentation

**Fig. 1:**
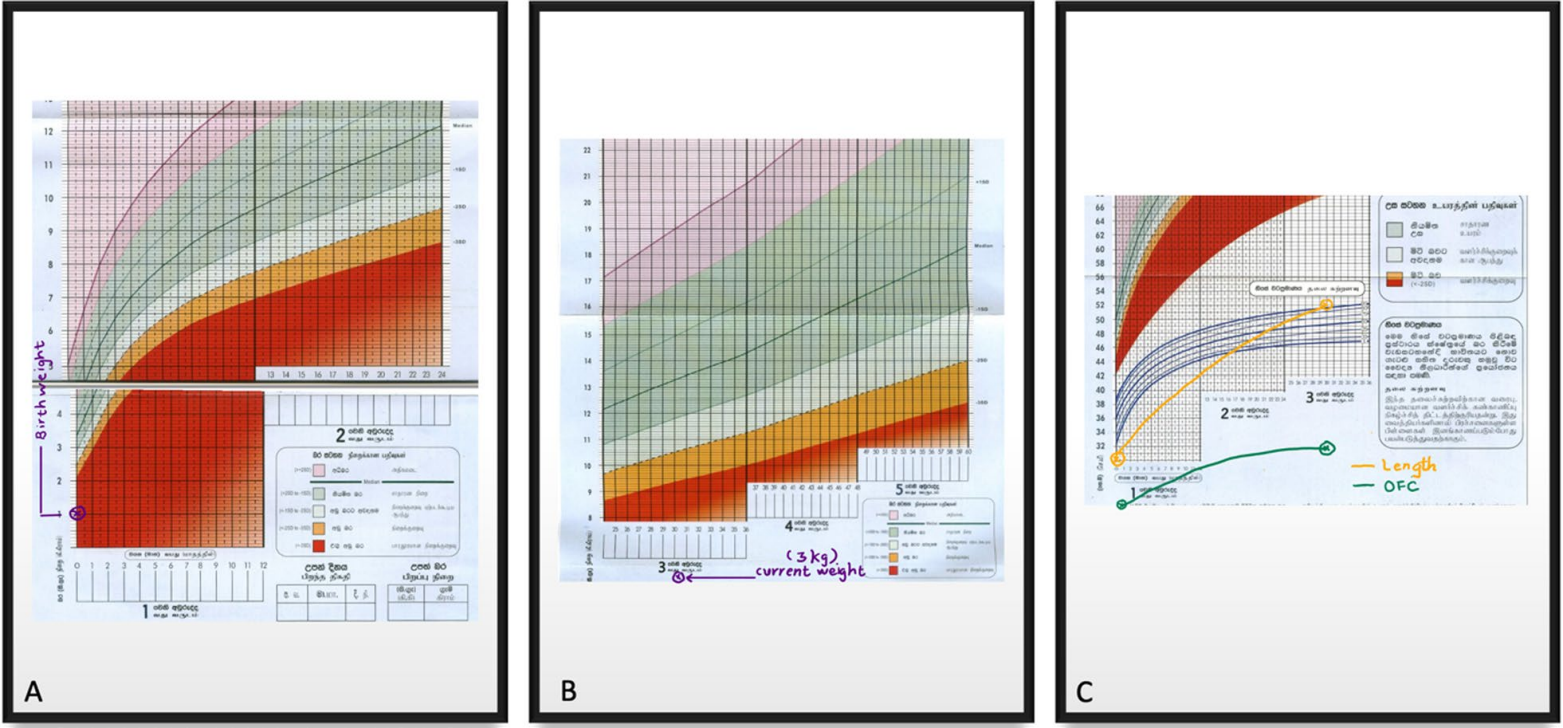
**A** Birth weight marked in WHO growth charts, **B** current weight marked in WHO growth charts, **C** length and OFC (birth and current measurements) marked in WHO growth charts

**Fig. 2:**
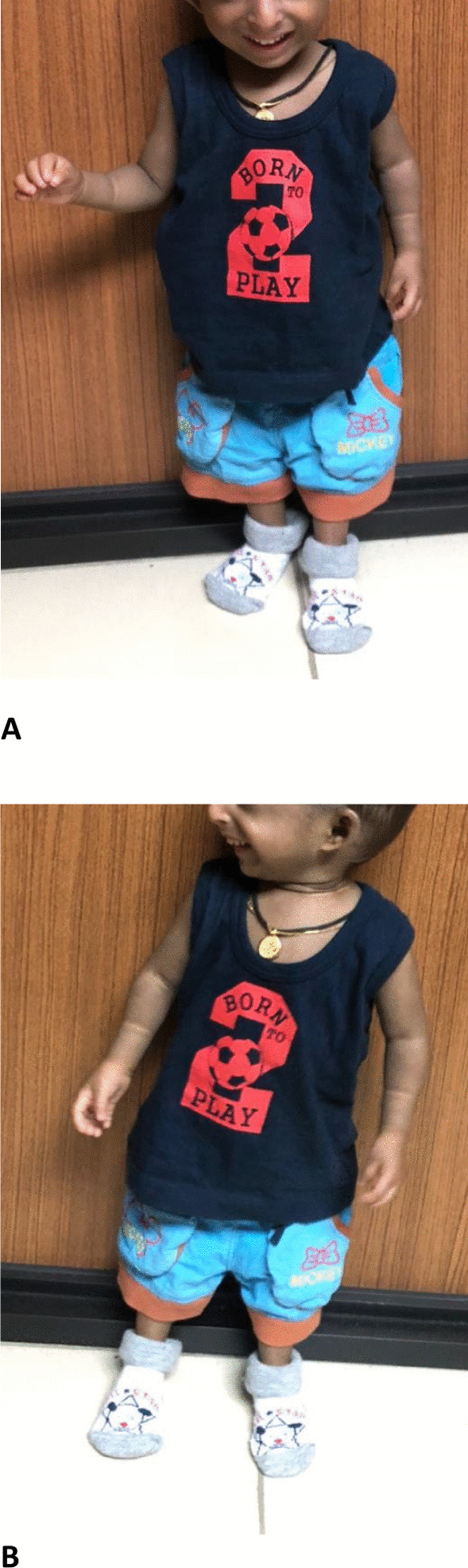
**A** frontal view of the face, **B** lateral view of the face

The proband is 2 years and 6 months old male. He is the only child to healthy nonconsanguineous parents of Sri Lankan origin. He was delivered when his mother was 24 years old, at 35 weeks of gestation via an emergency lower segment cesarean section due to severe oligohydramnios and marked fetal growth restriction. There was no history of antenatal bleeding, fetal decelerations, or any miscarriages before this pregnancy. His Apgar scores at 1 min and 5 min were 8 and 10 respectively. At birth, his anthropometric parameters were as follows; birth weight—1080 g, birth length—30 cm, and occipitofrontal circumference—24 cm. All parameters were well below—3SD in the standard WHO growth charts. He was admitted to the Special Care Baby Unit during the 1st week of life due to very low birth weight. From birth to 6 months, he had been extensively evaluated for poor weight gain. All recorded biochemical investigations were within the normal range. However, endocrine assessments related to growth were not performed due to financial constraints. His current growth parameters are shown in Fig. 1A–C which are also below—3SD. His developmental milestones remained age-appropriate since birth and there aren’t any symptoms suggestive of any food intolerances, metabolic or malabsorption syndromes. Despite optimal calorie intake, his growth velocity is constant. On examination, the following dysmorphisms were noted retrognathia, small ears, prominent nasal root with a large nose, microdontia, sparse scalp hair (Fig. 2A, B). He also has a characteristic high-pitched voice. Hypopigmented patches were noted in the upper limbs, but they did not follow the Blaschko lines. Limb examination revealed bilateral fifth finger clinodactyly. During other systems screening, echocardiography revealed a small ostium secundum atrial septal defect. An ultrasound scan of the abdomen revealed that the proband had markedly smaller normally functioning kidneys. His right and left kidneys measured 4.5 cm and 3.8 cm respectively. The normal range is 7.1 (6.8–7.4) cm for the right kidney and 7.0 (6.7–7.2) cm for the left kidney [9]. Therefore, the probands kidney is below the 1st percentile for the kidney length in cm according to age. The hip radiograph showed a poorly formed narrow pelvis with a flat acetabulum.

### Whole exome sequencing and bioinformatic analysis

**Fig. 3:**
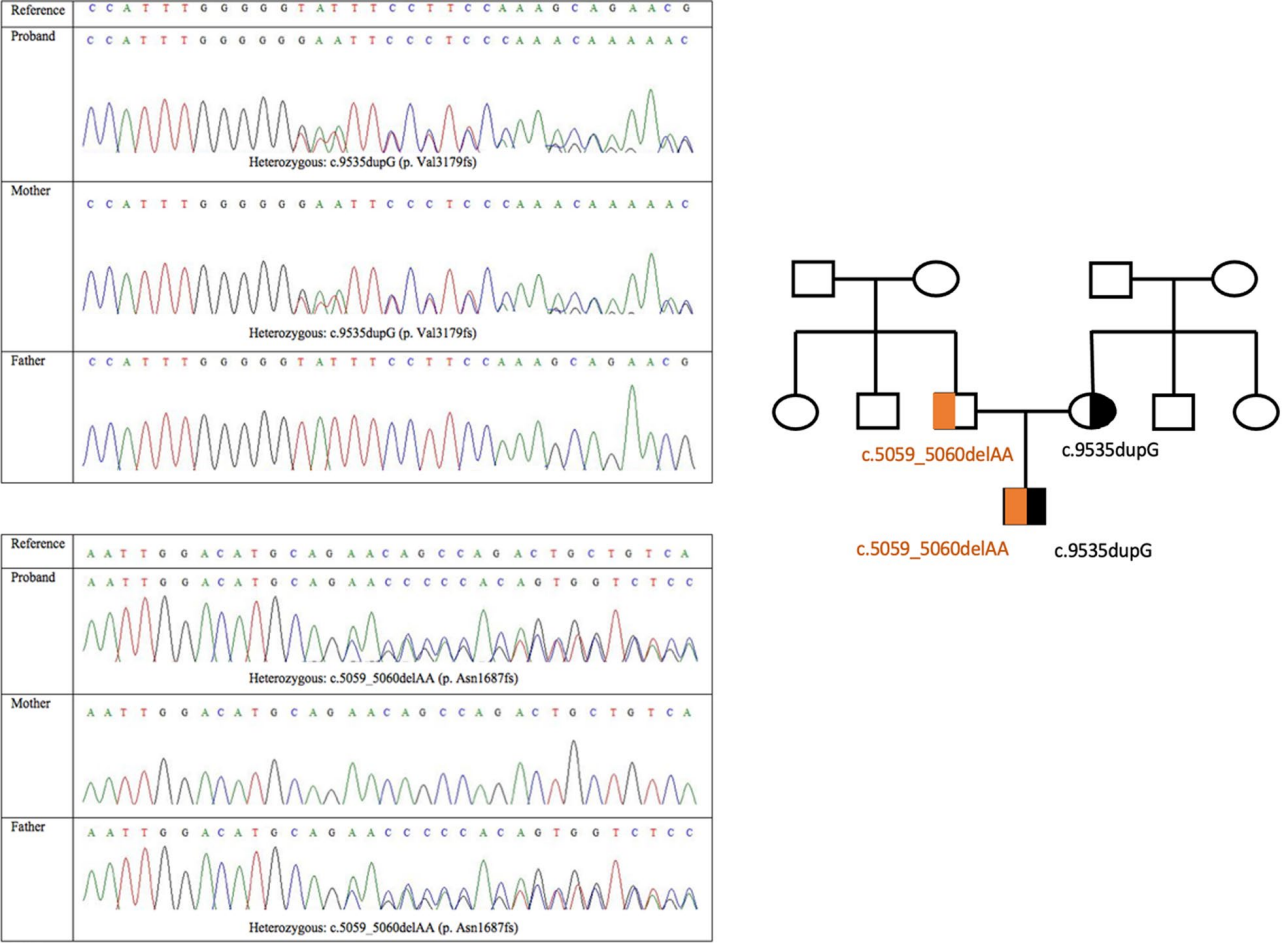
Sanger chromatograms of the *PCNT* gene variants in the proband (compound heterozygous) mother (heterozygous) and father (heterozygous) and the corresponding pedigree chart

Prior to performing Whole Exome Sequencing we obtained written informed consent from the proband’s parents under a protocol approved by the Ethics Review Committee of the Faculty of Medicine University of Colombo. Extraction of the genomic DNA from the blood leukocytes was done using the QIAamp DNA Mini Kit according to the manufacturer’s protocol. The SureSelectXT® Human(Mouse) All Exon V6 5190-886 kit was used in Illumina® NovaSeq® 6000 Next Generation Sequencer for Whole Exome Sequencing. An in-house bioinformatics pipeline was used to analyze the generated data. Aligning the paired-end sequencing data to GrCh37 human reference genome and variant calling was performed using the BWA-mem algorithm and Genome Analysis Tool Kit (GATK). Annotation of the generated variants calling format file was performed using SNP‐eff with the help of Refseq, clinical, and population frequency databases. Then a virtual gene panel consisting of genes that were known to cause skeletal dysplasia (Table 1) was used to filter out the variants relevant to the proband's phenotype. According to the standard ACMG guidelines (https://www.acgs.uk.com/media/11631/uk-practice-guidelines-for-variant-classification-v4-01-2020.pdf), benign variants were filtered out. In silico functional prediction tools (Mutation Taster, SIFT, PolyPhen2, and Provean) were used to predict the functional significance of the detected variants. Functional impact on the protein structure and conservation of the resided region were used to further scrutinize the variants. After filtration, results revealed a pathogenic compound heterozygous frameshift variant in the PCNT gene c.5059_5060delAA | p. Asn1687fs (novel variant) and c.9535dup (p. Val3179fs). On screening his parents, his mother and father were found to be heterozygous for the variants (Fig. 3).

**Table 1:** Skeletal dysplasia virtual gene panel

*ACAN*	*ACP5*	*ACVR1*	*ADAMTS10*	*ADAMTS17*	*AFF4*	*AGA*	*AGPS*
*AIFM1*	*ALPL*	*AMER1*	*ANKH*	*ANO5*	*ARCN1*	*ARSB*	*ARSE*
*ARSL*	*ASCC1*	*ASPM*	*ATR*	*B3GALT6*	*B3GAT3*	*B4GALT7*	*BGN*
*BMP1*	*BMP2*	*BMPER*	*BMPR1B*	*BPNT2*	*C2CD3*	*CA2*	*CANT1*
*CASR*	*CCDC8*	*CCN6*	*CDC45*	*CDC6*	*CDKN1C*	*CDT1*	*CENPJ*
*CEP120*	*CEP135*	*CEP152*	*CEP63*	*CFAP410*	*CHST14*	*CHST3*	*CHUK*
*CILK1*	*CLCN7*	*COG1*	*COL10A1*	*COL11A1*	*COL11A2*	*COL1A1*	*COL1A2*
*COL27A1*	*COL2A1*	*COL9A1*	*COL9A2*	*COL9A3*	*COMP*	*CREB3L1*	*CRTAP*
*CSF1R*	*CSGALNACT1*	*CSPP1*	*CTSA*	*CTSK*	*CUL7*	*CWC27*	*DDR2*
*DDRGK1*	*DHCR24*	*DIP2C*	*DLL3*	*DLX3*	*DMRT2*	*DNA2*	*DONSON*
*DVL1*	*DVL3*	*DYM*	*DYNC2H1*	*DYNC2LI1*	*EBP*	*EIF2AK3*	*ESCO2*
*EVC*	*EVC2*	*EXOC6B*	*EXOSC2*	*EXT1*	*EXT2*	*EXTL3*	*FAM20C*
*FAM46A*	*FAR1*	*FBN1*	*FGF23*	*FGF9*	*FGFR1*	*FGFR2*	*FGFR3*
*FIG4*	*FKBP10*	*FLNA*	*FLNB*	*FN1*	*FTO*	*FUCA1*	*FZD2*
*GALNS*	*GALNT3*	*GDF5*	*GDF6*	*GHR*	*GHRHR*	*GHSR*	*GJA1*
*GLB1*	*GMNN*	*GNAS*	*GNE*	*GNPAT*	*GNPTAB*	*GNPTG*	*GNS*
*GORAB*	*GPC6*	*GPX4*	*GSC*	*GUSB*	*GZF1*	*HES7*	*HGSNAT*
*HPGD*	*HSPG2*	*HYAL1*	*IARS2*	*ICK*	*IDS*	*IDUA*	*IFITM5*
*IFT122*	*IFT140*	*IFT172*	*IFT43*	*IFT52*	*IFT57*	*IFT74*	*IFT80*
*IFT81*	*IGF1*	*IGF2*	*IHH*	*IMPAD1*	*INPPL1*	*JAG1*	*KAT6B*
*KIAA0586*	*KIAA0753*	*KIF22*	*KL*	*KMT2A*	*LARP7*	*LBR*	*LEMD3*
*LFNG*	*LIFR*	*LIG4*	*LMNA*	*LMX1B*	*LONP1*	*LOXL3*	*LRP4*
*LRP5*	*LRRK1*	*LTBP2*	*LTBP3*	*MAFB*	*MAN2B1*	*MANBA*	*MAP3K7*
*MATN3*	*MBTPS2*	*MCM5*	*MCPH1*	*MEOX1*	*MESP2*	*MGP*	*MMP13*
*MMP14*	*MMP2*	*MMP9*	*MNX1*	*MSX2*	*MYH3*	*MYO18B*	*NAGLU*
*NANS*	*NBAS*	*NEK1*	*NEU1*	*NKX3-2*	*NOG*	*NOTCH2*	*NPPC*
*NPR2*	*NPR3*	*NSDHL*	*NSMCE2*	*NXN*	*OBSL1*	*OCRL*	*ORC1*
*ORC4*	*ORC6*	*OSTM1*	*P3H1*	*P4HB*	*PAM16*	*PAPSS2*	*PCGF2*
*PCNT*	*PCYT1A*	*PDE4D*	*PEX5*	*PEX7*	*PGM3*	*PISD*	*PKDCC*
*PLK4*	*PLOD2*	*PLS3*	*POC1A*	*POLR1A*	*POP1*	*POR*	*PPIB*
*PPP3CA*	*PRKAR1A*	*PTDSS1*	*PTH1R*	*PTHLH*	*PTPN11*	*PYCR1*	*RAB33B*
*RBBP8*	*RECQL4*	*RIPPLY2*	*RMRP*	*RNU4ATAC*	*ROR2*	*RSPRY1*	*RTTN*
*RUNX2*	*SC5D*	*SEC24D*	*SERPINF1*	*SERPINH1*	*SETBP1*	*SFRP4*	*SGSH*
*SH3PXD2B*	*SLC17A5*	*SLC26A2*	*SLC35D1*	*SLC39A13*	*SLCO2A1*	*SLCO5A1*	*SMAD4*
*SMARCAL1*	*SNRPB*	*SNX10*	*SOX9*	*SP7*	*SPARC*	*SQSTM1*	*SRCAP*
*SUCO*	*SULF1*	*TAB2*	*TAPT1*	*TBCE*	*TBX15*	*TBX3*	*TBX5*
*TBX6*	*TBXAS1*	*TCIRG1*	*TCTEX1D2*	*TCTN3*	*TGFB1*	*TMEM165*	*TMEM38B*
*TNFRSF11A*	*TNFRSF11B*	*TNFSF11*	*TRAPPC2*	*TREM2*	*TRIM37*	*TRIP11*	*TRMT10A*
*TRPS1*	*TRPV4*	*TTC21B*	*TUBGCP6*	*TYROBP*	*VAC14*	*VPS33A*	*WDR19*
*WDR34*	*WDR35*	*WDR60*	*WISP3*	*WNT1*	*WNT3*	*WNT3A*	*WNT5A*
*XRCC4*	*XYLT1*	*XYLT2*	*ZMPST*

### In-silico analysis

**Fig. 4:**
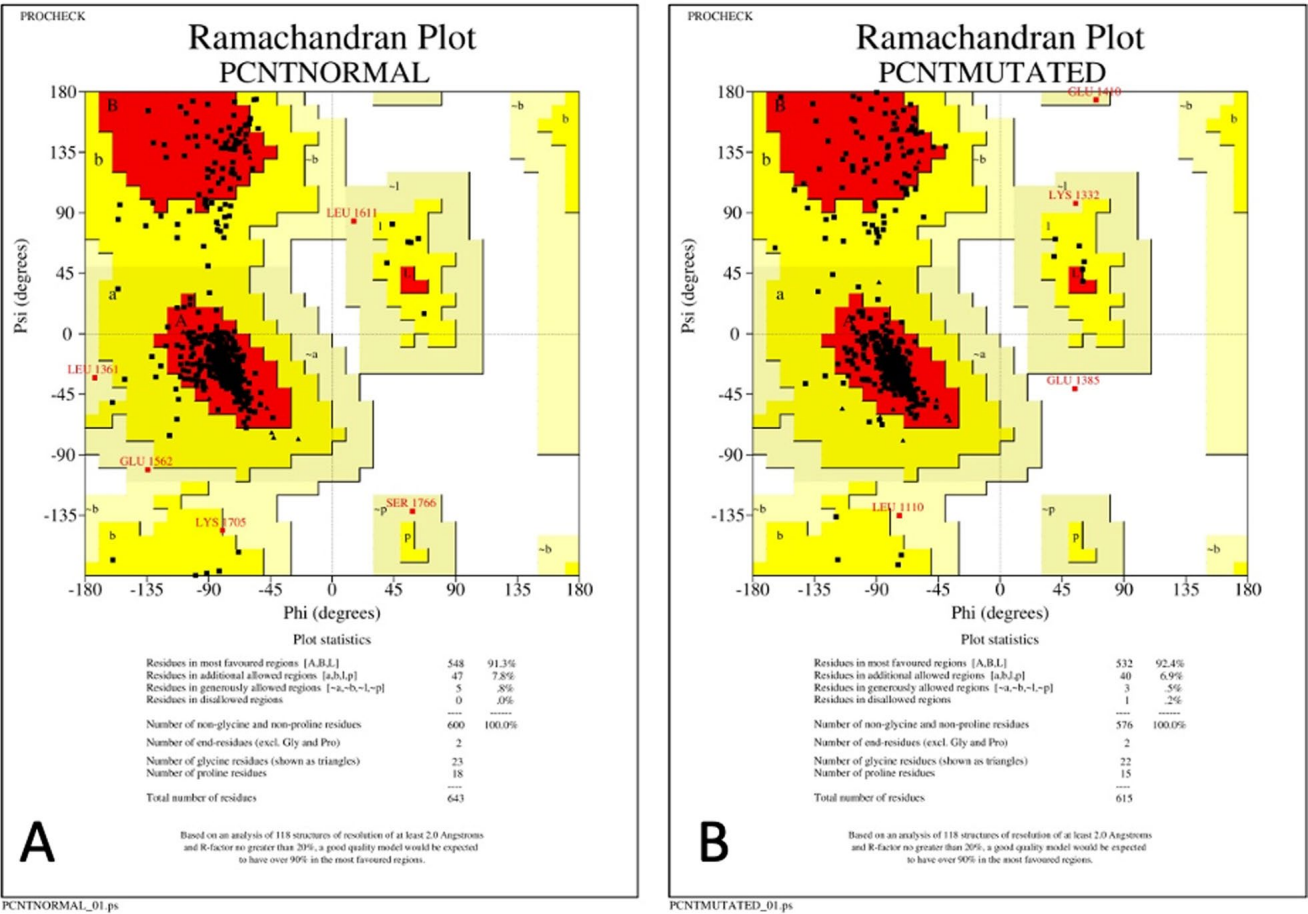
Ramachandran Plot showing the empirical distribution of amino acids observed in a **A** normal and **B** mutated PCNT protien structure for structure validation

**Fig. 5:**
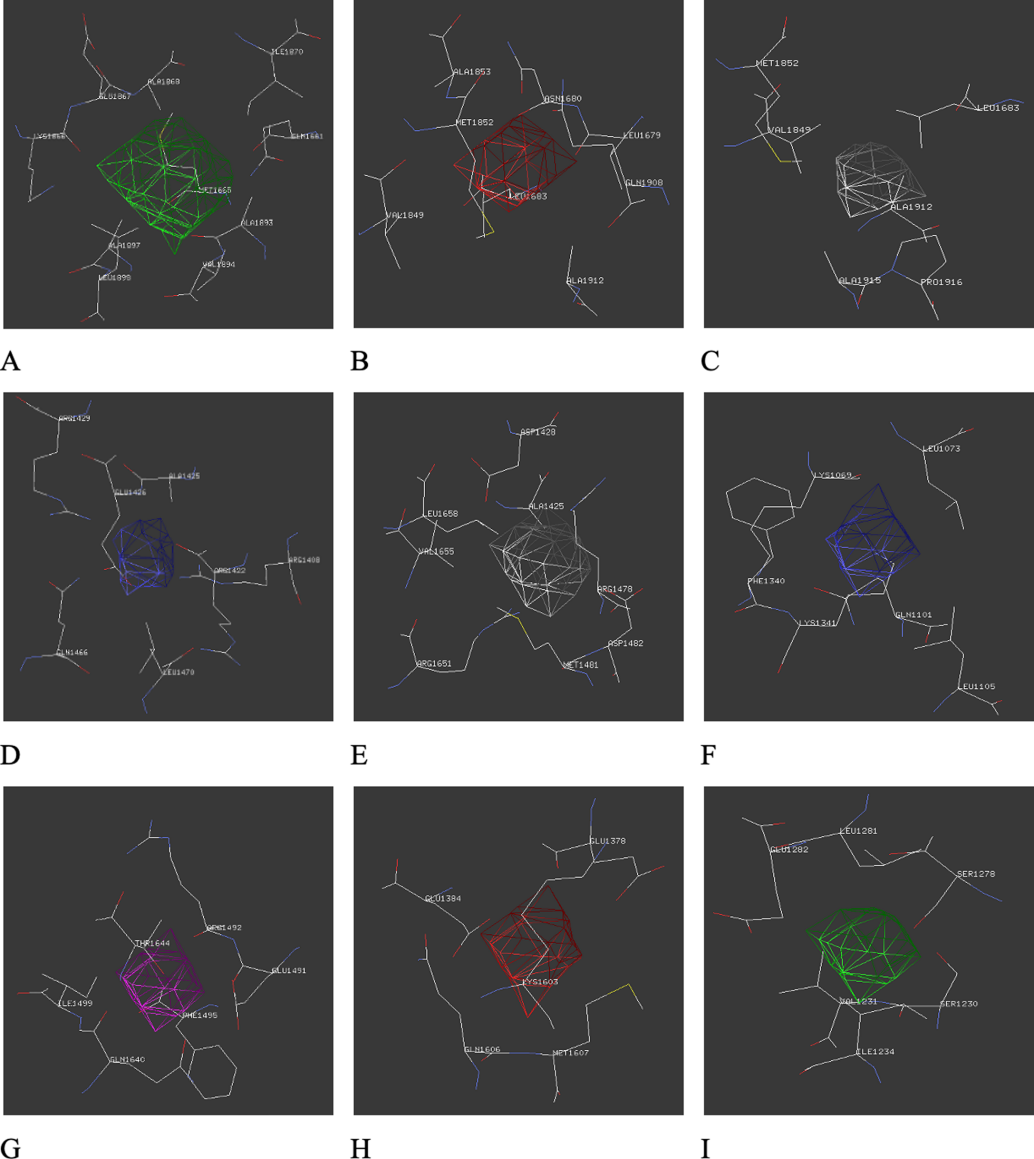
Amino acids present around the cavities of wild type (**A**–**D**) and mutated (**E**–**I**) PCNT protein

For genomic analysis of the present case, the FASTA sequence of the homo sapiens pericentrin B (PCNT2) mRNA, complete cds with GenBank ID: AF515282.1 of 10020 bp was downloaded from the NCBI nucleotide database (https://www.ncbi.nlm.nih.gov/nuccore/ AF515282). In the wild-type sequence, there were two adenine nucleotides at cDNA positions 5059 and 5060. Manual deletion of this nucleotide pair was carried out to generate the variant corresponding to the proband c.5059_5060delAA. Both mRNA sequences were submitted to the ORF tool to access the differences in the coding amino acids. We observed a change in the amino acid sequence owing to a shift in the reading frame. Thus, in the mutated protein at the 1687th position amino acid asparagine "N" was replaced by glutamine "Q". Furthermore, there was an appearance of TGA (i.e., termination codon) succeeding 10 triplet codons after the initial amino acid change. This resulted in premature termination of protein synthesis. The downloaded FASTA sequence of the wild-type protein (UniProtKB ID-O95613, PCNT_HUMAN) had 3336 amino acid residues and 3D modeling was carried out using the SPDBV offline software. Modeling of the wild type and mutated PCNT was done by homology approach by taking 1JQN as a template structure. According to the 3D models of the two proteins, the amino acid numbers were 643 and 615 respectively. We compared the number of non-glycine and non-proline residues which were 600 and 576 respectively. It was also observed that in the mutated protein one amino acid was found in the disallowed region thus contributing to an unstable tertiary structure (Fig. 4). There was also an increase in the number of cavities from 4 in the wildtype to 5 in the mutant protein with altered amino acid configurations around these cavities (Fig. 5).

## Discussion and conclusion

Herein we described the first genetically confirmed case of Microcephalic Osteodysplastic Primordial Dwarfism (MOPD) Type II in a patient of Sri Lankan origin. Based on the genetic diagnosis the parents were counselled and multidisciplinary shared care was arranged locally.

Growth monitoring was carried out with specific growth curves designed for this condition [10]. Thus, growth parameters of the proband were plotted between median and − 1 SD in MOPD Type II specific growth curves. A study conducted on 47 individuals with this condition revealed 64% of the study population was diagnosed with a vascular condition such as moyamoya and intracranial aneurysms or both. Additionally, vascular complications involving the renal arteries, coronaries, and external carotids were also reported in this group [2]. The proband had normal findings in the baseline ultrasound KUB and MRI brain. However, structural heart defects were reported which is a rare phenotype [11]. Due to the high risk of neurovascular manifestations in later life, routine surveillance was arranged as recommended in the literature. Proband will be followed up with multidisciplinary inputs by a cardiologist and neurologist. Furthermore, we observed bilaterally small kidneys in the proband, which was not described with MOPD Type II before. Yearly skeletal surveillance to identify hip pathologies such as hip dislocations and scoliosis is also essential, a narrow dysplastic hip with a flat acetabulum is a cardinal feature [12]. Oro dental findings such as microdontia, malformed teeth, short roots especially of the molars, tooth agenesis, and enamel hypoplasia are commonplace. Hence proper dental care and regular follow-up with a dentist are essential. Their also prone to endocrine abnormalities, mainly insulin resistance which should be anticipated after 5 years of age. Growth hormone therapy is not usually recommended considering the risk of scoliosis and the lack of evidence in the outcome.

MOPD Type II affects multiple organ systems this could reflect the spindle dysfunction caused by *PCNT* variants. Thus, loss of function of pericentrin is implicated in mislocalization of cellular proteins due to mitotic spindle defects causing missegregation of chromosomes, mitotic failure with eventual cell arrest, and cell death [13]. Pericentrin binds calmodulin expressed in the centrosomes, it contains a series of coiled-coil domains that interact with the microtubule nucleation component gamma-tubulin which is essential throughout the cell cycle. As confirmed by protein modeling approaches defects in the protein–protein interaction domains of PCNT could have contributed to the phenotype of MOPD Type II in our patient. According to the literature, there was a high incidence of cerebrovascular malformations when the last exons from 30 to 43 were involved [12]. The two variants identified in the proband c.9535dup (p.Val3179fs) resides in exon 43 and c.5059_5060delAA (p. Asn1687fs) resides in exon 27. The first variant is reported to cause non-sense-mediated decay of mRNA. It is present in population databases (rs747058622) at a very low frequency (G = 0.00002/5 (GnomAD_exomes) and G = 0.000033/4 (ExAC)) and is recorded as a likely pathogenic variant in the Clinvar database (Variation ID: 264920). The second variant has not previously been reported in population databases or clinical databases and represents a null allele in the *PCNT* gene for which loss-of-function is a known mechanism of MPOD II disease. In this study, the detection of biallelic variants in the *PCNT* gene confirmed the diagnosis of MOPD Type II and presented a new phenotype, thus expanding the phenotypic spectrum of *PCNT* variants associated with this condition.

## Data Availability

https://www.ncbi.nlm.nih.gov/clinvar/variation/1343402/?new_evidence=false; Accession: VCV001343402.1; https://www.ncbi.nlm.nih.gov/Traces/sra?run=SRR18395025.

## Abbreviations

MOPD: Microcephalic Osteodysplastic Primordial Dwarfism
PCNT: Pericentrin
SPDBV: Swiss-PdbViewer
I-TASSER: Iterative Threading ASSEmbly Refinement
NCBI: National Center for Biotechnology Information
